# Causal Effects of Ambient PM2.5 and Occupational Chemical Exposures on All‐Cause Dementia and Vascular Dementia: A Mendelian Randomization Study

**DOI:** 10.1002/alz70855_107401

**Published:** 2026-01-05

**Authors:** Faria Tavacoli, Jingchun Chen, Hayley Ho, Alice Lee

**Affiliations:** ^1^ University of Nevada Las Vegas, Las Vegas, NV, USA

## Abstract

**Background:**

Environmental evidence links environmental exposures, such as air pollution and occupational chemicals, to cognitive decline and dementia. However, their causality, particularly for all‐cause dementia (ACD) and vascular dementia (VAD) remains challenging due to residual confounding in observational studies. Leveraging Mendelian Randomization (MR), this study investigates whether genetically predicted exposure to fine particulate matter (PM2.5) and occupational chemicals causally influences dementia risk.

**Method:**

Genetic instruments for PM2.5 and workplace chemical/fume exposure were downloaded from UK Biobank genome‐wide association studies (GWAS). Outcome data for ACD and VAD were obtained from recent large‐scale GWAS. We applied multiple MR approaches including inverse variance weighted (IVW), MR Egger, and Weighted Median methods, to estimate causal effects. Sensitivity tests (e.g., Cochran's Q, MR‐Egger intercept, MR‐PRESSO) were used to assess heterogeneity, pleiotropy, and causal direction. Significance was defined as IVW *p* < 0.05 with consistent directional effects across sensitivity tests.

**Result:**

MR analyses showed that genetically predicted PM2.5 was associated with a 51.7% increased risk of ACD (OR = 1.517, 95% CI: 1.049–2.194; *p* = 0.027). Frequent workplace exposure to chemicals/fumes showed a 30‐fold higher risk of VAD (OR = 30.147, 95% CI: 1.583–574.204; *p* = 0.024), though the wide confidence interval warrants caution. Sensitivity analyses confirmed robustness with no evidence of heterogeneity (*p* = 0.143, 0.946), horizontal pleiotropy (*p =* 0.08, 0.09), and MR‐PRESSO (*p* = 0.145, 0.953).

**Conclusion:**

This MR study supports causal associations between PM2.5 air pollution and occupational chemical exposures in increasing dementia risk. The association with VAD highlights vascular pathways as potential mediators. While the magnitude of occupational risk requires replication due to wide confidence intervals, these findings highlight the urgent need for public health interventions to mitigate air pollution and occupational chemical exposure, ultimately reducing dementia risk and improving public health outcomes. Limitations of this study include the assumptions of MR, such as linearity and lifelong exposure effects, as well as the potential for unmeasured confounders in genetic instruments.

